# Practices and Strategies of Health Professionals during the COVID-19 Pandemic—Between Limitations and Opportunities

**DOI:** 10.3390/healthcare10101817

**Published:** 2022-09-21

**Authors:** Stinne Glasdam, Frode F. Jacobsen, Sigrid Stjernswärd

**Affiliations:** 1 Integrative Health Research, Department of Health Sciences, Faculty of Medicine, Lund University, Margaretavägen 1 B, S-222 41 Lund, Sweden; 2 Centre for Care Research, Western Norway University of Applied Services, P.O. Box 7030, N-5020 Bergen, Norway; 3 Health-Promoting Complex Interventions, Department of Health Sciences, Faculty of Medicine, Lund University, Margaretavägen 1 B, S-222 41 Lund, Sweden

The COVID-19 pandemic was declared as such in March 2020 [1] and took the world by surprise. It necessitated prompt and urgent action to prevent the further spread of the SARS-CoV-2 virus at local and global levels. International health authorities defined specific prevention measures, such as, quarantine, social distancing, and hand washing to prevent infections and minimise the spread of the virus [2]. The pandemic and associated management strategies came to affect individuals, groups, and whole societies at multiple levels [3,4], including health professionals [5]. These came to face new and challenging situations, which required massive efforts, engagement, dedication, and courage. 

During the current pandemic, frontline health professionals have stood on precarious grounds in an unknown situation and with continuous news feeds. Health professionals’ work situation was and is affected by the pandemic in multiple ways, for instance through an increased workload and elevated patient streams [6], increased absenteeism due to staff in quarantine [7], the handling of acute and unknown health conditions related to the infection with SARS-CoV-2 and related complications, additional tasks because of the relocation of colleagues to other units and related task shifts, the absence of relatives to support and care for patients, safety issues, and novel and constantly updated work routines [8]. Furthermore, the new and precarious COVID-19 situation affected frontline workers and their work in a range of different clinical settings. Examples include nursing staff working longer shifts in hospitals and facing burnout, fatigue, and insomnia [9], and nursing home staff experiencing additional stress related to sudden task shifts, longer shifts, and increased workload [7]. Further examples include mental health problems in staff working in home care services [10]. 

In addition, health professionals have faced massive information streams and situations in which there were no self-evident, evidence-based solutions. Clinical situations have encompassed difficulties navigating the contradictory needs for both closeness to and distance from patients [6], and finding time for catering to the increased need for information and follow-up of close family of patients. Like all of us, health professionals can be emotionally, physically, cognitively, and socially affected by an unpredictable future, including experiences of work-related and moral stress [11], feelings of anxiety, distress, insecurity, [12,13] post-traumatic reactions [14], and fear of infection [15], to name some aspects. This can have subsequent consequences on the health professionals’ professional, private, and social situations.

A crisis such as the pandemic places several constraints on the common practices of frontline health professionals, both in hospitals and in the primary health care sector [5]. Such constraints can also spill over to the health professionals’ private and social lives, where they may need to navigate and balance their professional and private roles, personas, and requirements in their everyday lives (see e.g., Glasdam et al., 2022 in the current Special Issue [16]). However, such sudden limitations can also open the door to creativity and new solutions. It may be that novel or already existing solutions, which are right up our street, are not used, and that they are not even recognised as possibilities. Glasdam et al.’s (2021) review [17], for instance, shows that different uses of social media are seen among nurses to gain clinically relevant information about the pandemic. Furthermore, limitations and crises can force us to think anew, to rethink existing routines (see e.g., Samuelson, 2022 in the current Special Issue [18]), and to address the strengths and weaknesses of existing healthcare and associated systems to develop adequate and effective solutions to both old and new problems and challenges. In addition, the pandemic demands new creative research strategies in frontline healthcare settings, while still ensuring robust, ethical, and safe methods, whilst being carried out at a distance (see e.g., Alhejaili et al., 2022 in the current Special Issue [19]).

This Special Issue focuses on how health professionals work between limitations and opportunities through the COVID-19 pandemic to handle the situation, with the potential to develop healthcare and health professionals’ practices in the future. This Special Issue of *Healthcare* was a call for original research, case studies, debate articles, reflections, and reviews on practices and strategies of health professionals created or exacerbated by the COVID-19 pandemic. This can relate to the development and adaptation of clinical guidelines; seeking new ways of working due to and related to the pandemic; clinical work with patients affected by COVID-19, relatives, colleagues, and other relevant stakeholders; experienced limitations in terms of existing knowledge and clinical routines, with more. These were only suggestions, to which the current issue was not necessarily limited. We welcomed all kinds of creative and innovative article suggestions in line with the topic at stake. 

## Data Availability

Not applicable.
